# Intracellular localization of Crimean-Congo Hemorrhagic Fever (CCHF) virus glycoproteins

**DOI:** 10.1186/1743-422X-2-42

**Published:** 2005-04-25

**Authors:** Sebastian Haferkamp, Lisa Fernando, Tino F Schwarz, Heinz Feldmann, Ramon Flick

**Affiliations:** 1 University of Texas Medical Branch, Department of Pathology, Center for Biodefense and Emerging Infectious Diseases, 301 University Boulevard, Galveston, Texas, 77555-0609 USA; 2 Special Pathogens Program, National Microbiology Laboratory, Health Canada, CA-R3E 3R2 Winnipeg, Canada; 3 Stiftung Juliusspital Wuerzburg, 97070 Wuerzburg, Germany; 4 Department of Medical Microbiology, University of Manitoba, 543-730 William Avenue, Winnipeg, R3E 0W3 Canada

## Abstract

**Background:**

Crimean-Congo Hemorrhagic Fever virus (CCHFV), a member of the genus *Nairovirus*, family *Bunyaviridae*, is a tick-borne pathogen causing severe disease in humans. To better understand the CCHFV life cycle and explore potential intervention strategies, we studied the biosynthesis and intracellular targeting of the glycoproteins, which are encoded by the M genome segment.

**Results:**

Following determination of the complete genome sequence of the CCHFV reference strain IbAr10200, we generated expression plasmids for the individual expression of the glycoproteins G_N _and G_C_, using CMV- and chicken β-actin-driven promoters. The cellular localization of recombinantly expressed CCHFV glycoproteins was compared to authentic glycoproteins expressed during virus infection using indirect immunofluorescence assays, subcellular fractionation/western blot assays and confocal microscopy. To further elucidate potential intracellular targeting/retention signals of the two glycoproteins, GFP-fusion proteins containing different parts of the CCHFV glycoprotein were analyzed for their intracellular targeting. The N-terminal glycoprotein G_N _localized to the Golgi complex, a process mediated by retention/targeting signal(s) in the cytoplasmic domain and ectodomain of this protein. In contrast, the C-terminal glycoprotein G_C _remained in the endoplasmic reticulum but could be rescued into the Golgi complex by co-expression of G_N_.

**Conclusion:**

The data are consistent with the intracellular targeting of most bunyavirus glycoproteins and support the general model for assembly and budding of bunyavirus particles in the Golgi compartment.

## Background

Crimean-Congo hemorrhagic fever virus (CCHFV) is a member of the genus *Nairovirus*, one of five genera in the family *Bunyaviridae *[1]. Bunyaviruses are enveloped particles with a tripartite, single stranded RNA genome of negative polarity [2-4]. The three genome segments encode four structural proteins: the RNA-dependent RNA polymerase (L protein) is encoded by the large (L) segment, the glycoproteins (GN and GC; previously referred to as G1 and G2) are encoded by the medium (M) segment, and the nucleocapsid protein (N) is encoded by the small (S) segment [2-4].

The virus glycoproteins are likely to play an important role in the natural tick-vertebrate cycle of the virus as well as for the high pathogenicity in humans. Indeed, a highly variable mucin-like region at the amino terminus of the CCHFV glycoprotein precursor has recently been identified, a unique feature of nairoviruses within the family *Bunyaviridae *[5]. A similar serine-threonine-rich domain has been associated with increased vascular permeability and development of hemorrhages in Ebola hemorrhagic fever [6].

The *Nairovirus *genus includes 34 described viruses and is divided into seven different serogroups [1]. Only three viruses are known to cause disease: CCHFV, Dugbe virus, and Nairobi sheep disease virus. CCHFV is an arthropod-borne pathogen and the causative agent of a serious form of hemorrhagic fever [7-9] with mortality rates ranging from 15 to 60% [10-17]. The virus is endemic in parts of Africa, Southeastern Europe and Asia as far east as western China [16,18,19]. The geographic distribution of CCHFV infections corresponds most closely with the distribution of *Hyalomma *ticks, suggesting their principal vector role [18,20,21]. *Hyalomma *ticks normally feed on a variety of livestock (sheep, goats, cattle, and ostriches), large wild herbivores, hares, and hedgehogs, which can become infected with CCHFV [13,18,22,23]. In contrast to human infections, infection in these animals generally results in inapparent or subclinical disease but generates viremia levels capable of supporting virus transmission to uninfected ticks [10,18,21,23-25]. Transmission to humans occurs either by bites from infected ticks or direct contact with blood or tissues of infected livestock. Nosocomial infections are common [26] and represent a major problem in health care institutions [27].

The widespread geographical distribution of CCHFV, its ability to produce severe human disease with high mortality rates, and fears about its intentional use as a bioterrorism agent  makes CCHFV an extremely important human pathogen and a worldwide public health concern. Case management and intervention strategies would greatly benefit from knowledge of the biology and pathogenesis of the virus.

Recently, the expression strategy and biosynthesis of the CCHFV glycoproteins have been studied in more detail including the identification of precursor cleavage sites and the determination of the exact N termini of the two major cleavage products, GN and GC [5,28]. SKI-1, also responsible for the proteolytic processing of the Lassa virus glycoprotein precursor [29], has been identified as the cellular protease responsible for the processing step that generates the N-terminus of mature GN. Another yet unidentified protease is required for GC processing. However, the exact C-terminus of GN could not yet been determined. Two cleavage sites have been predicted for this processing step, one at amino acid position 808 (RKLL) and the other at 940/944 (KKRKK) favouring the cellular proteases SKI-1 and furin, respectively, as the responsible proteases [28].

Bunyaviruses are known to bud from Golgi membranes and the budding site seems to be defined by an retention of the glycoproteins GN and GC at that particular site [3,4]. From a number of studies which have addressed the mechanisms of Golgi targeting and retention, one can conclude that the N-terminal located glycoprotein appears to carry the appropriate signal(s) [30-40] So far, no studies have investigated Golgi targeting and retention of nairovirus glycoproteins.

In this study we cloned the complete M segment ORF of CCHFV, strain IbAr10200, into different expression plasmids. Expression and intracellular localization of the glycoproteins GN and GC were studied and compared to glycoproteins generated by virus infection. Using recombinant fusion proteins between the green fluorescence protein (GFP) and CCHFV glycoproteins, the Golgi targeting/retention signal could be mapped to a hydrophobic region within the cytoplasmic domain of the GN protein.

## Results

### Sequence determination of the full-length CCHFV M segment

The complete M segment nucleotide sequences of two different sources of CCHFV, strain IbAr10200, was determined and compared to previously published sequences [GenBank: U39455]. Several nucleotide changes resulting in amino acid changes in the glycoprotein precursor were identified (Table 1). In two different CCHF viral RNA samples eight amino acid changes and two silent nucleotide changes could be detected. Four additional amino acid changes were found in sample #2 as well as four silent nucleotide changes not leading to any amino acid alteration. CCHFV RNA sample #1 showed two additional unique amino acid changes.

**Table 1:** Sequence comparison of available CCHFV IbAr10200 M segment sequences

**CCHF IbAr10200**	Nucleotide changes compare to U39455 (vRNA position*)	Amino acid changes compare to U39455*
Samples 1 and 2	83: C → T	1671: Gly → Arg
	713: T → C	1461: Ser → Gly
	1621: T → C	1158: Glu → Gly
	2263: G → T	944: Thr → Lys
	2926: C → T	723: Arg → Lys
	2964: A → G	710: – (silent)
	3512: C → T	528: Val → Ile
	3550: A → G	515: Phe → Ser
	4044: G → T	350: – (silent)
	4981: T → G	38: His → Pro
Sample 2	3425: T → C	557: Asn → Asp
	3427: C→ A	556: Cys → Phe
	3429: G → A	555: – (silent)
	3435: T → A	555: – (silent)
	3441/42: GT → AG	551: Asp → Ala
	3444: A → T	550: – (silent)
	4247: G → A	282: – (silent)
	4610: T → G	162: Thr → Pro
Sample 1	2760/61: TA → AT	778: Ile → Asn
	4684: G → A	137: Ser → Phe

* based on [GenBank: U39455] position numbering

**Figure 1:**
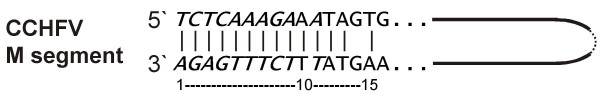
**Schematic presentation of the predicted base-paired ends of the CCHFV M vRNA segment**. The first nine nucleotides as well as nucleotides at position 11 at both RNA ends are highly conserved within the three genome segments (bold, italics), whereas the first thirteen nucleotides at the 3' and 5' vRNA ends are inverted complementary and can form base-paired terminal regions.

Furthermore, we determined the sequences of the exact ends of the M segment using an RNA ligation approach. Beside constructs with nucleotide deletions due to RNA degradation prior to RNA ligation several full-length sequences were determined, demonstrating the expected homologous RNA ends compare to the CCHF S and L segments (Fig. 1). Especially the first and last nine nucleotides of the CCHF M vRNA segment showed high complementarity to the L and S segment ends (bold and italicized nucleotides in Fig. 1), confirming their role as important *cis*-acting elements for RNA polymerase binding [41,42].

### Expression of CCHFV glycoproteins

**Figure 2:**
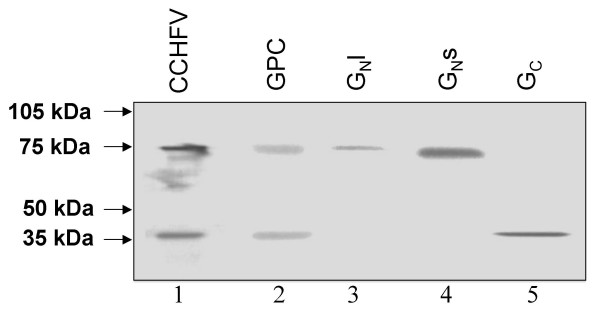
**Western blotting of CCHFV glycoproteins after transfection of different expression plasmids**. BHK-21 cells were either infected with CCHFV (lane 1) or transfected with individual CCHFV glycoprotein expression plasmids (lanes 2–5). Twenty-four hours post infection/transfection cells were harvested and cell lysate used for western blotting, using immune serum specific against CCHFV IbAr10200. Protein bands with expected sizes were detected and confirmed successful expression of CCHFV glycoproteins.

Based on the recently published N-terminal sequence determination of mature CCHFV glycoproteins [5] and using the above described determined CCHFV M segment sequence (sample #1), expression plasmids for both glycoproteins G_N _and G_C _as well as for the glycoprotein precursor (GPC) were generated. Since the C-terminus of CCHFV G_N _has not yet been determined ([28]; S. Nichol: pers. communication) two constructs were generated containing an N-terminal Influenza HA-tag for detection: pCMV CCHF G_N _"short" (G_N_s) and pCMV CCHF G_N _"long" (G_N_l). Glycoprotein expression was first analyzed by immunoblot using CCHFV-specific polyclonal or HA-tag antibodies. The CCHF full-length glycoprotein precursor construct (pCAGGS CCHFV GPC) was successfully expressed and correctly processed into the cleavage fragments G_C _and G_N _(Fig. 2, lane 2). Molecular weights (G_C_, 37 kDa and G_N_, 75 kDa) as determined by immunoblot analysis were in accordance with those of the G_C _and G_N _expressed in CCHFV-infected VeroE6 cells (Fig. 2, lane 1). CMV-driven HA-G_N_s and HA-G_N_l expression resulted in a protein of approximately 75 kDa (Fig. 2, lanes 3 and 4), similar to authentic G_N _glycoprotein seen in CCHFV-infected cells (Fig. 2, lane 1). Expression of chicken β-actin-driven G_C _resulted in a product of approximately 37 kDa, again similar to G_C _expression in CCHV-infected cells (Fig. 2, lane 5). The data demonstrates that each glycoprotein can be authentically expressed individually from separate plasmids (e.g., pCMV G_N_s, pCMV G_N_l and pCAGGS G_C_) as well as from a clone encoding the GPC precursor (pCAGGS GPC) using polyclonal CCHFV-specific and HA-tag antibodies (Fig. 2). Expression could also be confirmed using CCHFV-specific G_C _and G_N _antipeptide antibodies which were kindly provided by S. Nichol, CDC) (data not shown).

### Intracellular localization of CCHFV glycoproteins

**Figure 3:**
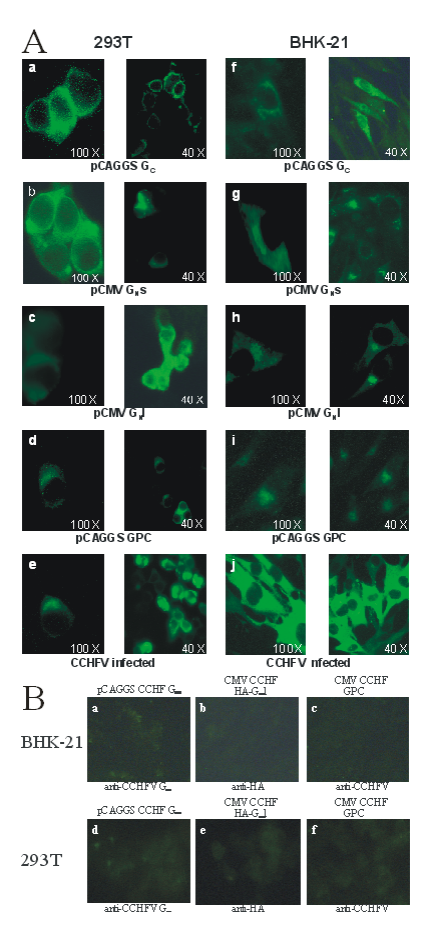
**Indirect immunofluorescence assays of CCHFV glycoproteins after transfection of different expression plasmids**. BHK-21 and 293T cells were transfected with CCHFV glycoprotein expression plasmids. Twenty-four hours post transfection cells were fixed and stained with CCHFV-specific or anti-HA antibodies. **A: **Cells fixed with methanol/acetone allow analyses of intracellular proteins. Polyclonal antibodies against CCHFV G_C _were used for G_C _detection (a, f). CCHFV G_N _expression from two different CMV-driven expression plasmids was analyzed using anti-HA tag antibodies (b, c, g, h). G_N _and G_C _expressed from the GPC were studied using polyclonal anti-CCHFV antibodies (d, i) as well as specific antipeptide antibodies against G_N _and G_C _(data not shown). CCHFV-infected cells served as controls (e, j). **B: **Cells fixed with paraformaldehyde were analyzed for CCHFV G expression on cellular surfaces. G_C _was stained using anti-CCHFV G_C _antibodies, G_N _with anti-HA tag, and mature CCHFV proteins derived from the GPC with anti-CCHFV antibodies. No clearly visible staining correlates with no detectable surface expression.

Indirect immunofluorescence assays (IFA) were initially performed to analyze the cellular localization of CCHFV glycoproteins. For this, different CCHFV glycoprotein expression plasmids were individually transfected into BHK-21 or 293T cells and 24 to 48 h post transfection the cells were fixed with acetone/methanol or paraformaldehyde for intracellular (Fig. 3A) or surface immunofluorescence analysis (Fig. 3B), respectively. HA-specific monoclonal antibodies were used to detect the two forms of individually expressed N-terminal HA-tagged G_N _(Fig. 3A: b, c, g, h) and CCHFV G_C_-specific antibodies were used to monitor β-actin promoter-driven G_C _expression products (Fig. 3A: a, f). In addition, a CCHFV-specific antiserum was used to detect G_N _and G_C _expression from full-length glycoprotein precursor construct pCAGGS GPC (Fig. 3A: d, i) as well as in CCHFV-infected cells (Fig. 3A: e, j). In all cases G_N _and G_C _were detected intracellular but never on the cell surface (Figs. 3A and 3B). Mock-infected and -transfected cells were used as negative controls (data not shown). Two different cell lines were used to exclude artificial cell type-specific localization pattern of CCHFV glycoproteins.

**Figure 4:**
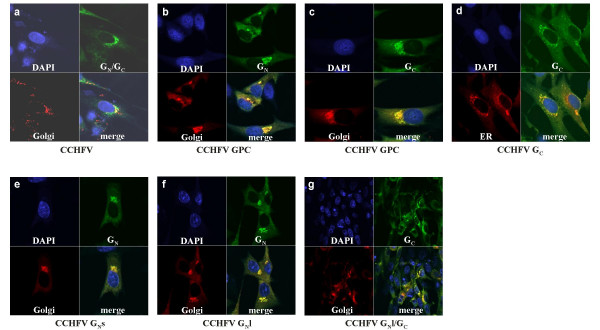
**Intracellular co-localization studies of CCHFV glycoproteins and cellular compartment markers**. BHK-21 cells were either infected with CCHFV or transfected with individual CCHFV glycoprotein expression plasmids. Twenty-four hours post infection/transfection cells were fixed with methanol/acetone and subsequently an indirect immunofluorescence assay was performed. DAPI-stained (for nucleus localization), Golgi or ER compartment stained and CCHFV G expression pictures were taken individually and subsequently merged to study the intracellular CCHFV G localization. **a: **CCHFV-infected cells stained for CCHF G_N _and G_C _proteins; **b and c: **CCHFV GPC expression plasmid-transfected cells stained for G_N _or G_C_, respectively; **d: **Cells transfected with CCHFV G_C _expression plasmid stained with G_C_-specific antibodies and co-stained with ER compartment marker calreticulin; **e: **CCHFV G_N_s-transfected cells stained with HA tag-specific antibodies; **f: **CCHFV G_N_l-transfected cells stained with HA tag-specific antibodies; **g: **CCHFV G_C _expression pattern after co-transfection of CCHFV G_N_l and G_C_.

In a next step we tried to specify the intracellular localization of CCHFV GN and GC glycoproteins expressed from plasmids encoding either the individual glycoproteins or the precursor GPC. Intracellular staining pattern of CCHV-infected cells as well as cells expressing the CCHFV precursor GPC revealed a Golgi complex staining pattern independent of the antibodies used for detection of the individual glycoproteins (Fig. 3A: d, e, i, j). Subsequently, we analyzed the intracellular localization of individually expressed GN and GC. Whereas individually expressed GN showed a Golgi complex localization (Fig. 3A: b, c, g, h), individually expressed GC accumulated in the perinuclear region of the cell indicative of ER localization (Fig. 3A: a, f). Confirmation for these results were achieved by co-immunofluorescence analyzed on a confocal microscope using CCHFV glycoprotein-specific or HA-specific antibodies and either antibodies directed against the ER-specific marker molecule calreticulin or direct staining of the Golgi region with BODIPY-TR C5 ceramides (Fig. 4). Again, CCHFV GN expression from the two expression plasmids pCMV GNs and pCMV GNl overlapped with Golgi staining (Fig. 4: e, f), whereas GC expression overlapped with that of calreticulin (Fig. 4: d). However, co-expression of both CCHFV glycoproteins either from the glycoprotein precursor plasmid or from simultaneous transfection of the two expression plasmids resulted in Golgi targeting of both glycoproteins (Fig. 4: c, g) strongly indicating that GN drives the Golgi localization and that GC needs to interact with GN in order to be transported out of the ER.

**Figure 5:**
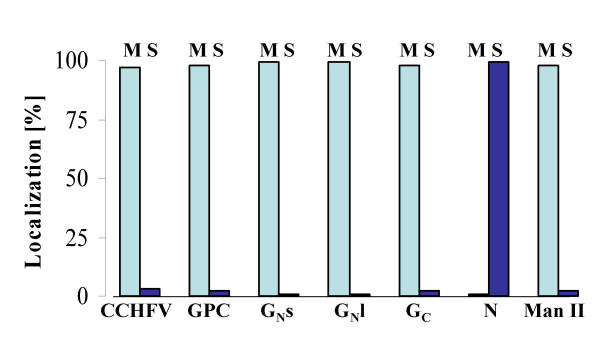
**Subcellular fractionation studies of expressed CCHFV glycoproteins**. CCHFV-infected or CCHFV G expression plasmid-transfected BHK-21 cells were used for a subcellular fractionation study to determine if CCHFV G proteins are membrane associated. The results shown are summarized from two independent experiments. M: membrane fraction, S: soluble fraction, N: CCHF nucleoprotein, Man II: Mannosidase II. For quantification individual protein bands were analyzed and compared using the software Quantity One (BioRad).

To further strengthen the association of CCHFV glycoproteins with intracellular membrane-containing compartments such as ER and Golgi complex, we performed subcellular fractionation experiments. This method allows the separation of soluble proteins from membrane-associated proteins. CCHFV-infected cells were used for comparison (Fig. 5: lane 1). As expected all expressed CCHFV glycoproteins were exclusively found in the pellet fractions, which contain membrane-associated proteins. This confirms the intracellular localization of these proteins with membrane structures and together with the co-immunofluorescence data confirms either ER or Golgi localization (Fig. 5: lanes 2 to 5). To evaluate the described approach control experiments using either the soluble CCHFV N proteins or the Golgi marker Mannosidase II were performed. As expected CCHF N protein was exclusively found in the soluble fraction, whereas the Golgi marker protein was only detected in the membrane-associate fraction.

### Signals for intracellular targeting of CCHFV glycoproteins

**Figure 6:**
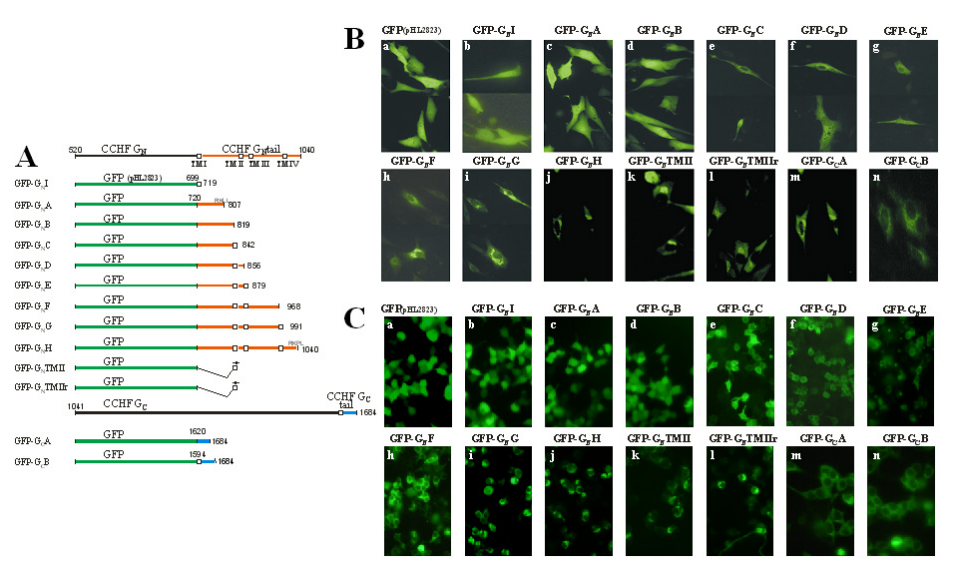
**GFP-CCHFV G fusion proteins to identify Golgi and ER localization signals**. BHK-21 and 293T cells were transfected with individual GFP-CCHFV G fusion proteins. Twenty-four hours post transfection cells were analyzed via UV microscopy. **A: **Schematic presentation of different GFP-CCHFV G fusion proteins. Green labeled parts represent the GFP protein, red and blue labeled parts show the fragments of the CCHFV G cytoplasmic tails C-terminal fused to the GFP gene. White boxes symbolize the predicted hydrophobic transmembrane domains (TM). Numbers represent the amino acid position from the CCHFV GPC; **B: **BHK-21 cell analyses; **C: **293T cell analyses.

After determining the intracellular localization of the CCHFV glycoproteins, we next were interested to determine the signals for intracellular targeting. For this, we generated GFP-fusion proteins containing different fragments of the G_C _or G_N _proteins attached to GFP. On the basis of published data obtained with other bunyaviruses we expected Golgi localization signals rather within the transmembrane or cytoplasmic domains than in the ectodomain [3,4]. A CMV-driven GFP expression plasmid (pHL2823, Flick and Hobom, unpublished) was used as a cloning vector for fusing different regions of the CCHFV glycoproteins to the C-terminus of the GFP. Firstly, the different PCR-amplified G_N _cytoplasmic domain fragments (Table 2) were cleaved with *Bsm*BI and inserted into pHL2823 after *Bam*HI/*Xba*I endonuclease treatment. In an alternative approach a signal peptide (Ig κ-chain signal of the pDisplay vector) was fused to the GFP N-terminus to allow entry into the secretory pathway. Secondly, the G_N _transmembrane domain (TM I) was inserted using a hybridized oligonucleotide linker (RF372/RF373: GATCCTTTGGCTATGT AATAACCTGCATACTTTGCAAGGCCATTTTTTACTTGTTAATAATTGTTGGATAAT/ CTAGATTATCCAACAATTATTAACAAGTAAAAAATGGCCTTGCAAAGTATGCAGGTTATTACATAGCCAAAG). The expression of the resulting constructs GFP-G_N_A, GFP-G_N_B, GFP-G_N_C, GFP-G_N_D, GFP-G_N_E, GFP-G_N_F, GFP-G_N_G, GFP-G_N_H, and GFP-G_N_I (Fig. 6A) was first verified by immunoblot (data not shown). All constructs expressed GFP-fusion proteins of expected sizes and were subsequently used in co-localization studies. For this two different cell lines (BHK-21 and 293T), for comparison purposes, were transfected with the different plasmid DNAs and GFP fluorescence localization was analyzed using UV-microscopy.

**Table 2:** Features and construction details of different GFP-CCHFV G fusion proteins

**Construct**	**Oligo-nucleotide primer**	**Restriction endonuclease**	**PCR-fragment length**	**Glycoprotein fragment [nt] Fragment length**
GFP-G2I	RF372 RF373	*Bam*HI *Xba*I	72 bp Oligonucleotide linker	3177-3113 64 nt
GFP-G2A	RF364 RF363	*Bsm*BI	296 bp	3114-2854 290 nt
GFP-G2B	RF364 RF365	*Bsm*BI	332 bp	3114-2818 296 nt
GFP-G2C	RF364 RF366	*Bsm*BI	401 bp	3114-2749 365 nt
GFP-G2D	RF364 RF367	*Bsm*BI	443 bp	3114-2707 407 nt
GFP-G2E	RF364 RF368	*Bsm*BI	512 bp	3114-2638 476 nt
GFP-G2F	RF364 RF369	*Bsm*BI	779 bp	3114-2371 743 nt
GFP-G2G	RF364 RF370	*Bsm*BI	848 bp	3114-2302 812 nt
GFP-G2H	RF364 RF371	*Bsm*BI	995 bp	3114-2155 959 nt
GFP-G1A	RF361 RF362	*Bam*HI *Xba*I	210 bp	411-220 191 nt
GFP-G1B	RF362 RF378	*Bgl*II *Xba*I	288 bp	489-220 69 nt

* based on [GenBank: U39455] position numbering

The fusion protein GFP-G_N_I, containing the TM I of CCHF G_N _was expressed in the cell cytoplasm in both used cell lines (Figs. 6B and 6C: b) similarly to GFP expressed from the basic vector pHL2823 (Figs. 6B and 6C: a). In case of the signal peptide-containing GFP fusion protein a diffuse staining consistent with the distribution throughout the secretory system was observed (data not shown). Based on this result we conclude that the transmembrane domain TM I does not contain any intracellular targeting signal.

**Figure 7:**
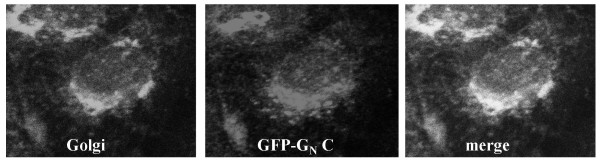
**Intracellular co-localization analysis of GFP-CCHFV G fusion proteins**. 293T cells were transfected with a GFP-CCHFV G_N _fusion protein construct encoding 123 amino acids from the G_N _cytoplasmic tail (GFP G_N_C). Twenty-four hours later cells were fixed with methanol/acetone. A subsequent indirect immunofluorescence using the Golgi compartment marker mannosidase II was performed and analyzed via UV microscopy. The merged picture clearly demonstrates the Golgi localization of the fusion protein.

The fusion proteins GFP-G_N_A and GFP-G_N_B showed a similar cytoplasmic expression pattern (Figs. 6B and 6C: c, d). GFP-G_N_A contains the first 87 amino acids from the cytoplasmic domain including the RKLL motif at position 808, which is a predicted protease cleavage motif for generating the C-terminus of the mature G_N _protein, whereas GFP-G_N_B has 99 amino acids fused to the GFP C-terminus, corresponding to the first G_N _cytosolic tail fragment, which is followed by a second hydrophobic region predicted as a potential transmembrane domain 2 (TM II) (compare Fig. 6A). Interestingly, the fusion proteins GFP-G_N_C, GFP-G_N_D, GFP-G_N_E, GFP-G_N_F, GFP-G_N_G, and GFP-G_N_H, which contain longer fragments of the predicted G_N _cytoplasmic domain including additional predicted hydrophobic transmembran regions (Fig. 6A), showed an increased level of similarity to the intracellular pattern of G_N_l, which contained the entire G_N _cytoplasmic domain up to the determined mature G_C _start (Figs. 6B and 6C: e-j). The switch from a diffuse staining pattern to a Golgi complex localization is caused by the addition of TM II to the first 99 amino acids of the cytoplasmic domain resulting in GFP-fusion proteins containing 122 amino acids of the predicted G_N _cytoplasmic domain (Fig. 6A). These results demonstrate that the Golgi targeting signal is not located within the first 99 amino acids of the G_N _cytoplasmic domain. However, the addition of an additional hydrophobic 23 amino acid stretch (TM II) result in a co-localization of the GFP-fusion protein with the Golgi complex marker mannosidase II (Fig. 7), demonstrating that a Golgi localization signal is located within the predicted TM II.

The Golgi localization signal was further analyzed with two more GFP-fusion proteins containing only the 23 amino acids from the predicted TM II directly fused to the C-terminus of GFP. To determine if a specific primary sequence within TM II was recognized as a signal or rather the hydrophobic character of this region was crucial to target GFP to the Golgi complex, the 23 amino acids were fused in two different orientations (Fig. 6A). BHK-21 (Fig. 6B) and 293T (Fig. 6C) cells were transfected with these constructs and GFP expression and intracellular localization were analyzed. Both GFP-fusion proteins showed specific Golgi complex localization demonstrating that TM II contains a Golgi localization signal and that the orientation of the primary amino acid sequence is not important for GFP translocation (Figs. 6B and 6C: k, l). GFP fusion proteins containing either the predicted G_C _TM (GFP G_C_A) or cytoplasmic domain (GFP G_C_B) showed perinuclear staining, suggesting ER localization (Figs. 6B and 6C: m, n).

**Figure 8:**
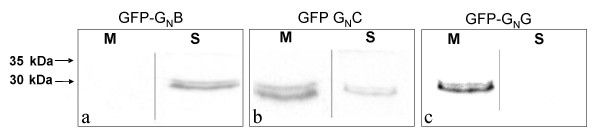
**Subcellular fractionation studies of expressed GFP-CCHFV G fusion proteins**. Expression plasmids for GFP-CCHFV glycoprotein fusion protein were transfected into BHK-21 cells and used for a subcellular fractionation study to determine if fusion proteins containing different parts from the CCHFV G_N _cytoplasmic domain are soluble or membrane associated. A GFP-specific antibody was used for the immunoblot. Representative results from three constructs are shown. M: membrane fraction, S: soluble fraction.

Subsequent analyses of expressed GFP-G_N _fusion proteins with subcellular fractionation approaches were performed to confirm the association of the fusion proteins with cellular membranes and to demonstrate the transition of intracellular localization from a diffuse cytoplasmic to a Golgi complex region pattern (Fig. 8). For this, membrane-associated cellular proteins were separated from soluble proteins and the different fractions analyzed via immunoblot using GFP-specific antibodies. As expected, constructs GFP G_N_I, GFP G_N_A, and GFP G_N_B, containing only the TM I, 87 or 99 amino acids from the predicted G_N _cytoplasmic domain, respectively, were only detected in the soluble fraction (Fig. 8a; only shown for G_N_B), whereas GFP G_N_C and constructs with longer parts from the G_N _cytoplasmic domain including the TM II region were detected mainly in the pellet fraction containing membrane-associated proteins (Fig. 8b). Constructs with longer fragments of the G_N _cytoplasmic domain, including additional TM regions, were exclusively detected within the pellet fraction (e.g., GFP G_N_G; Fig. 8c). These results confirmed our previous findings that the addition of G_N _TM II results in a change of intracellular protein localization and seems to mediate targeting to Golgi membranes.

## Discussion

Enveloped viruses, which do not acquire their lipoprotein coat by budding through the plasma membrane bud at internal membranes, including the inner nuclear membrane (herpesviruses;[43], the ER (flaviviruses and rotaviruses; [44,45], the intermediate compartment (ERGIC) (coronaviruses and poxviruses; [46,47], and the Golgi complex (rubellaviruses, coronaviruses, and bunyaviruses; [4,46,48]. Usually, the accumulation of the viral surface proteins in the specific intracellular compartment determines the assembly and budding site of the virus. This intracellular accumulation is dependent on certain compartment-specific retention or retrieval signals.

For almost all bunyaviruses assembly and budding takes place in the Golgi region [4,46,48]. However, so far no common motifs could be identified for signals within bunyaviral glycoproteins resulting in Golgi targeting and accumulation. Indeed, even the locations of such signals within bunyaviral glycoproteins are different. For the phlebovirus Uukuniemi (UUK), a Golgi retention signal could be identified within the membrane-proximal half (aa1040) of the 81 aa long cytoplasmic domain of G_N _[30,31,38]. In contrast, for the phlebovirus Punto Toro, such signals were mapped to the transmembrane domain (TM) and the adjacent amino acids of the G_N _cytoplasmic domain [36,37]. A similar localization was recently described for the Golgi retention signal in the G_N _of the phlebovirus Rift Valley Fever (RVF) virus G_N _[34]. Notable, for the Old World hantavirus Hantaan (HTN) it was reported that the conformation of the G_N_/G_C _complex might play a more important role for Golgi accumulation than an actual primary sequence motif [39].

While extensive studies have been performed regarding intracellular budding sites and glycoprotein accumulation for members of the genera *Orthobunyavirus*, *Phlebovirus*, *Hantavirus *and *Tospovirus *[30,32,34-36,39,40], nothing is known for members of the genus *Nairovirus*. Here we demonstrated, for the first time, that the CCHFV G_N _protein is membrane associated and contains a Golgi localization motif. In addition we have mapped this signal to a hydrophobic region (TM II) within the predicted cytoplasmic tail [5]. Co-expressed GN and GC result in a specific Golgi accumulation and co-localization using specific Golgi markers, whereas individual expressed GC is retained in the ER. These results imply that the two CCHFV glycoproteins have to interact and form hetero-oligomers for a proper Golgi transport of G_C_.

GFP-fusion proteins containing different portions of the CCHF G_N _glycoprotein allowed mapping of the Golgi targeting sequence within the cytoplasmic domain. Interestingly, we located the signal downstream of the predicted protease cleavage site RKLL at position 808 of the CCHFV precursor GPC, responsible for generating the C-terminus of the mature G_N _protein [5,28], implying that this cleavage site might not be used during the maturation process of G_N_. Furthermore, we could demonstrate that the addition of only the hydrophobic region from the predicted TM II within the G_N _cytoplasmic domain targeted a GFP-fusion protein to the Golgi complex. This shows that the 23 amino acids of TM II are sufficient and necessary for targeting GFP to the Golgi region, whereas the first 99 amino acids from the cytoplasmic domain and the TM I domain do not contribute to Golgi targeting.

The results obtained from the GFP-G_N _fusion proteins seem contradictory to the studies with the G_N _expression plasmid. IFA data combined with confocal microscopy co-localization studies of cells transfected with G_N_s expression plasmids demonstrated a clear Golgi complex staining (Fig. 3A: b, g; Fig. 4e). Since G_N_s contains only the first 87 amino acids of the predicted cytoplasmic domain without the predicted TM II sequence, we expected that the corresponding GFP-fusion protein GFP-G_N_A would show similar intracellular localization. However, the diffuse staining throughout the cytoplasm of transfected cells demonstrates that the first 87 amino acids are not sufficient to target the GFP to the Golgi complex (GFP-G_N_A; Figs. 6B and 6C: c). A possible explanation for this discrepancy is the existence of a second Golgi localization signal located within the G_N _ectodomain. Such a signal would be the reason for the Golgi localization pattern of G_N_s, whereas GFP-G_N_C and fusion proteins containing longer fragments of the predicted G_N _cytoplasmic domain localize to the Golgi region because of a Golgi localization signal located in TM II.

CCHFV G_C _protein expressed by its own retained in the ER and did not relocate into the Golgi complex. Interestingly, similar to all described G_C _proteins of phleboviruses CCHFV G_C _proteins also contain a lysine-based ER retrieval signal (KKXX; [49] within the predicted cytoplasmic domain. In case of single expressed G_C _protein this signal is most likely responsible for the ER localization of the protein, even though GFP-CCHF G_C_A fusion proteins containing only the predicted TM showed perinuclear staining pattern (Figs. 6B and 6C: m). However, co-expression with G_N _protein leads to interaction between these two proteins most likely resulting in masking of the ER retrieval signal and an accumulation of the heterodimer in the Golgi complex, due to the Golgi retention signal(s) located on G_N _(Fig. 3A: d, e, i, j; Fig. 4g). A similar phenomenon with conflicting transport/targeting signals was previously described for the rubellavirus E1 and E2 proteins [48,50,51].

## Conclusion

In conclusion, we were able to express CCHF G_N _and G_C _glycoproteins individually as well as from the precursor GPC. G_N _could be localized to the Golgi compartment, whereas G_C _was found in the ER. Co-expression of both proteins resulted in Golgi rescue of G_C_, indicating that proper interaction between G_N _and G_C _is important for transportation of the heterodimer out of the ER. The potential Golgi targeting signal could be localized to a hydrophobic region within the cytoplasmic domain in the G_N _protein. Furthermore, our results suggest that additional signals could be localized within the G_N _ectodomain.

Further characterization of the CCHFV G_N _Golgi retention signals could provide helpful information to understand the proteolytic cleavage event(s) of the GPC and the glycoprotein maturation process. The different CCHFV G expression plasmids might show also useful for the generation of virus-like particles (VLPs) as well as for identification of interaction sites between the viral glycoproteins and the ribonucleoproteins.

The identification of the potential budding site(s) of nairoviruses and the detailed analysis of the Golgi localization signal of the CCHFV G_N _protein will allow subsequent studies for targeting the glycoprotein accumulation during the development of antiviral strategies or even for rational vaccine design.

## Methods

### Cells and virus

BHK-21 (baby hamster kidney), 293T (human embryonic kidney), VeroE6 (African green monkey kidney) and SW13 cells (human adenocarcinoma cells)(American Type Culture Collection) were grown on plastic dishes in Glasgow (BHK-21), Eagle's minimal essential (293T, VeroE6), or Leibovitz L15 (SW13) medium, respectively, supplemented with 5 to 10% fetal calf serum, 2 mM L-glutamine, 100 IU of penicillin/ml, and 100 μg of streptomycin/ml (Invitrogen). The CCHFV, strain IbAr10200, isolated in 1970 from ticks (*Hyalomma excavatum*) in Nigeria (Sokoto), kindly provided by Special Pathogens Branch, Centers for Disease Control and Prevention, Atlanta (T. G. Ksiazek), was used for all experiments. The CCHFV stocks were prepared on SW13 cells by infection of T162 cell culture flasks with a 1:100 dilution. Supernatant was collected three days post infection (p.i.), clarified from cell debris by low speed centrifugation (3,000 × *g*, 10 min, 4°C), and aliquots were stored in liquid nitrogen. Virus titers were determined either by plaque assay or 50% tissue culture infectious dose assay (TCID50).

### Sequence determination of the full-length CCHFV M segment

Total RNA was isolated 7 days post infection from VeroE6 cells infected with CCHFV (1:1000 dilution of virus stock; 10^-3 ^pfu, RNA sample #1). Additional CCHFV RNA was kindly provided by J. Smith, USAMRIID, Alphavax, Durham, N.C. (RNA sample #2). CCHFV specific M segment vRNA or cRNA molecules were reverse transcribed using the primers CCHF M1 (TCTCAAAGAAATAGTGGCGGCACGCAGTC) or CCHF M2 (TCTCAAAGAAATACTTGCGGCACGTCAGT) for the reverse transcription reaction, respectively. The resulting cDNA molecules were used as templates for subsequent PCR reactions producing overlapping PCR fragments covering the entire CCHFV M segment. PCR products were inserted into pCR4 using the TOPO TA cloning kit (Invitrogen). Prior to sequence determination, positive clones were screened by PCR technology (primer TOPO F: AGCTCGGATCCACTAGTAACG and TOPO R: ATGCTCGAGCGGCCGCCAGTG) and restriction enzyme digest (*Eco*RI). For vRNA and cRNA-based constructs three of the cloning plasmids were sequenced using primers specific for the M segment ORF. The sequence results were aligned to the genebank sequence U39455 using the Align Plus 5 program of the Clone Manager Professional Suite 6 (Scientific & Educational Software). Determined nucleotide exchanges and the corresponding amino acid differences are listed in Table 1. For sequence determination of the M segment ends CCHFV specific vRNA and cRNA molecules were ligated using T4 RNA ligase (Roche) prior to the reverse transcription reaction (vRNA: M32: AGAACCAGAGGCCTGTTCAA, cRNA: M33: AAGGTGTCTGTGCCGGTTGT). Subsequent PCR amplification with primers CCHF M34 (AATACTAGTCTAAT CCACTGGCTGGTGTT) and M35 (AATGAATTCTGCCGAACTGTTCTCTAC) generated fragments containing both segment ends. PCR products were inserted into pCR4 (Invitrogen) for direct sequence determination as described above. In total, 12 cRNA/mRNA and 37 vRNA clones were analyzed using T7 and T3 promoter-specific primers.

### CCHFV glycoprotein expression plasmids

Based on the recently published N-terminal sequence determination of mature CCHFV glycoproteins [5], expression plasmids for both glycoproteins were generated. In case of the CCHFV G_N _two constructs were generated since the C terminus of the mature GN is not yet experimentally determined: pCMV CCHF G_N _"short" (G_N_s) contains the G_N_-ORF from pos. 519 to 807, preceding the predicted C-terminal cleavage site RKLL at position 808 (44). pCMV CCHF G_N _"long" (G_N_l) consists of pos. 519 to 1040 extending the G_N_-ORF to the experimentally determined N-terminal end of GC. The PCR fragments (G_N_s: RF346/352, G_N_l: RF353/352) were inserted after *Bsm*BI endonuclease treatment into pDisplay (Invitrogen) previously digested with *Bgl*II/*Xma*I digest, resulting in CMV-driven (human cytomegalovirus immediate early promoter and enhancer) expression plasmids for CCHFV G_N_. The Ig κ-chain signal peptide sequence and the hemagglutinin A (HA) epitope of the pDisplay vector were used for correct intracellular processing and detection, respectively. The CCHFV G_C _was PCR-amplified using primers CCHF M9 (AGTTGGTCTAGCCAATGTGTG) and RF351 (AATCGTCTCAAATTCATGGAGAC AGACACACTCCTGCTATGGGTACTGCTGCTCTGGGTTCCAGGTTCCACTGGTGACTTCCTAGATAGTACAGCTAAAGGCATG) (pos. 1041 to 1684).

*Bsm*BI- and *Xho*I-restricted PCR fragments were inserted into the plasmid pCAGGS/MCS [52] prior digested with *Eco*RI/*Xho*I digest, resulting in a chicken β-actin-driven expression plasmid for CCHFV G_C_. For correct intracellular processing of the CCHFV G_C _we inserted the Ig κ-chain signal peptide of the pDisplay vector via forward oligonucleotide primer RF351.

Different expression strategies (CMV-, chicken β-actin-driven) were used for the different CCHFV glycoproteins to yield maximum expression levels.

### Transfection

CCHFV glycoprotein expression plasmid DNA was transfected into subconfluent BHK-21 or 293T cells (3 × 10^6^) using 2 to 4 μg of the respective plasmid and 8 μl of liposome plus buffer (LipofectAMINE PLUS; Life Technologies, Invitrogen) mixed in serum-free MEM and incubated for 15 min at room temperature. After addition of 12 μl of liposome reagent, incubation was continued for a further 15 min. The cells were incubated at 37°C with the DNA-Lipofectamine mixture for 3 h. To determine the efficiency of transfection, plasmid pHL2823, expressing enhanced GFP (EGFP) under the CMV immediate early promoter and enhancer (R. Flick and G. Hobom, unpublished), was transfected similarly. After further incubation for 20–24 h in MEM containing 2% FCS, the transfected cells were fixed and CCHFV glycoprotein expression levels determined using indirect immunofluorescence assays (IFA).

### Indirect immunofluorescence assay

293T or BHK-21 cells grown on coverslips within a 6-well dish were transfected as described above. After 20 to 44 h, cycloheximide (final concentration of 0.18 mM) was added when indicated to inhibit further protein synthesis. The cells were incubated for an additional 2 to 5 h and then washed with phosphate buffer saline (PBS, pH 7.5) and fixed in methanol:acetone (50:50, V/V) for 20 min at -20°C. Permeabilization was omitted by fixation with paraformaldehyde when surface-expressed proteins were to be detected. After fixation, cells were washed with PBS and blocked for at least 30 min with PBS containing 5 % bovine serum albumin (BSA). Poly- or monoclonal antisera were diluted in PBS containing 1 % BSA and incubated for 1 h at room temperature. After several washes with PBS, goat anti-rabbit or mouse immunoglobulin secondary antibodies conjugated to fluorescein isothiocyanate (FITC) or tetramethyl rhodamin isothiocyanate (TRITC) were incubated with the cells for 45 to 60 min at room temperature. Procedures were repeated for double labeling with a different antiserum and fluorescent probe, and at the end of the procedure the slides were washed with PBS overnight.

Intracellular localization of the glycoproteins G_N _was determined by co-localization with commercially available organelle-specific fluorescent dyes (Molecular Probe Inc., Oregon, USA): BODIPY-TR C5 ceramide was selected as an indicator of the Golgi region. In addition Golgi (mannosidase II; Sigma) and ER-specific (Calreticulin; Sigma) monoclonal or polyclonal antibodies were used.

### Confocal Microscopy

Sample preparation and immunocytochemical staining were the same as for wide-field fluorescence microscopy. The fluorescence staining patterns were analysed with a ZEISS LSM 510 UV META laser scanning confocal microscope (Jena, Germany) equipped with a Coherent Enterprise II 81 mW Argon UV laser, a Lasos 30 mW Argon laser, and 5 mW HeNe laser. Images were acquired with a C-apochromat 63/1.2 corr. water-immersion lens. FITC-stained proteins were imaged with excitation at 488 nm and with a 505 to 530 nm bandpass emission filter. Golgi marker BODIPY-TR C5 ceramide were imaged with excitation at 543 nm and with a 570 to 655 nm bandpass emission. DAPI-stained DNA was imaged with excitation at 364 nm and emission through a 385 to 470 bandpass filter. Merged pictures for analysis of intracellular co-localization were generated using Zeiss LSM Image Brower 3.2 software.

### Membrane Fractionation

Alkaline carbonate extraction was performed on BHK-21 cells 24–48 h post transfection. The protocol described in Current Protocols in Cell Biology Online, John Wiley & Sons, Inc. was followed. Briefly, BHK-21 cells were transfected with individual constructs as described before. At 24 to 48 h post transfection, supernatant was removed and cells were washed three times with PBS followed by an additional washing step with 100 ml NaCl. Occasionally, the transfected cells would detach from the plate thus, the non-adherent cells were isolated between washes by microcentrifugation (2 min at 1000 × *g*). Remaining cells were scraped (adherent) or resuspended (non-adherent) into 1 ml of ice-cold 100 mM sodium carbonate, pH 11.5 and homogenized (five strokes) in a 2 ml Dounce homogenizer. The homogenate was then incubated for 30 min on ice and 1 ml of sodium carbonate was added to attain the necessary volume for subsequent ultracentrifugation (2 ml). The homogenate was then centrifuged for 60 min at 50.000 rpm using a TLS-55 rotor (Beckman) at 4°C. Following centrifugation, the supernatant was transferred to a fresh tube and concentrated three to five times. The pellet was resuspended in 250 μl of sodium carbonate. Pellet and supernatant fractions were then mixed with 4× SDS-PAGE sample buffer containing β-mercaptoethanol and run on SDS-PAGE. Protein gels were then transferred to PVDF transfer membrane (Amersham Biosciences) using a Trans-blot SD semi-dry transfer apparatus (Bio-Rad). Proteins were subsequently visualized by immunoblot.

### Western Blot

Following transfer, the blot was blocked overnight in 5 % skim milk + 0.1 % Tween. The following morning, the blot was washed three times with PBS/0.1 % Tween then incubated with the primary antibody, e.g. anti-GFP (Oncogene) at a 1:2000 dilution in PBS for 1 h at room temperature with rocking. The blot was then washed three times with PBS/0.1 % Tween and incubated with the secondary antibody, goat anti rabbit HRP (Sigma) at a 1:30.000 dilution in PBS for 1 h at room temperature with rocking. The blot was then washed with PBS/0.1 %Tween three times, followed by three washes with PBS. Proteins were visualized using the ECL+plus Western Blotting Detection system (Amersham Biosciences).

## Authors' contributions

SH carried out the described cloning work, confocal microscopy studies and the GFP-fusion protein analysis. LF carried out the membrane fractionation. TS revised the manuscript critically. HF helped to draft the manuscript and revised it critically. RF designed the study and coordinated and helped to draft the manuscript. All authors read and approved the final manuscript.

## References

[B1] van Regenmortel MHV, Fauquet CM, Bishop DML, Carstens EB, Estes MK, Lemon SM, Maniloff J, Mago MA, McGeoch DJ, Pringle CR, Wicknen RB (2000). 7th report of the International Committee of Taxonomy of Viruses. Virus Taxonomy.

[B2] Elliott RM, Schmaljohn CS, Collett MS (1991). Bunyaviridae genome structure and gene expression. Curr Top Microbiol Immunol.

[B3] Schmaljohn CS, Le Duc JW, Collier LH (1998). Bunyaviridae. Topley and Wilson's Microbiology and Microbial Infections.

[B4] Schmaljohn CS, Hooper JW, Knipe DMPMH (2001). Bunyaviridae: the viruses and their replication. Fields virology.

[B5] Sanchez AJ, Vincent MJ, Nichol ST (2002). Characterization of the glycoproteins of Crimean-Congo hemorrhagic fever virus. J Virol.

[B6] Yang ZY, Duckers HJ, Sullivan NJ, Sanchez A, Nabel EG, Nabel GJ (2000). Identification of the Ebola virus glycoprotein as the main viral determinant of vascular cell cytotoxicity and injury. Nat Med.

[B7] Casals J (1969). Antigenic similarity between the virus causing Crimean hemorrhagic fever and Congo virus. Proc Soc Exp Biol Med.

[B8] Chumakov MP, Sokolov AACMPKAA (1945). A new tick-borne disease-Crimean hemorrhagic fever. Crimean Hemorrhagic Fever (Acute Infectious Capillary Toxicosis).

[B9] Chumakov MP (1974). Contribution to 30 years of investigation of Crimean haemorrhagic fever. Med Virol.

[B10] Gonzalez JP, Camicas JL, Cornet JP, Wilson ML (1998). Biological and clinical responses of west African sheep to Crimean-Congo haemorrhagic fever virus experimental infection. Res Virol.

[B11] Leshchinskaya EV (1965). Clinical picture of Crimean hemorrhagic fever (in Russian). (in English: NAMRU3-1856).

[B12] Oldfield EC, Wallace MR, Hyams KC, Yousif AA, Lewis DE, Bourgeois AL (1991). Endemic infectious diseases of the Middle East. Rev Infect Dis.

[B13] Shepherd AJ, Swanepoel R, Cornel AJ, Mathee O (1989). Experimental studies on the replication and transmission of Crimean-Congo hemorrhagic fever virus in some African tick species. Am J Trop Med Hyg.

[B14] Swanepoel R, Porterfield JS (1995). Nairovirus Infections. Exotic Viral Infections.

[B15] Swanepoel R, Gill DE, Shepherd AJ, Leman PA, Mynhardt JH, Harvey S (1989). The clinical pathology of Crimean-Congo hemorrhagic fever. Rev Infect Dis.

[B16] Watts DMTGKKJLHH, Monath TP (1988). Crimean-Congo hemorrhagic fever. The arboviruses: epidemiology and ecology.

[B17] Yen YC, Kong LX, Lee L, Zhang YQ, Li F, Cai BJ, Gao SY (1985). Characteristics of Crimean-Congo hemorrhagic fever virus (Xinjiang strain) in China. Am J Trop Med Hyg.

[B18] Hoogstral H (1979). The epidemiology of tickborne Crimean-Congo hemorrhagic fever in Asia, Europe and Africa (Review). J Med Entomol.

[B19] Schwarz TF, Nitschko H, Jager G, Nsanze H, Longson M, Pugh RN, Abraham AK (1995). Crimean-Congo haemorrhagic fever in Oman. Lancet.

[B20] Logan TM, Linthicum KJ, Bailey CL, Watts DM, Moulton JR (1989). Experimental transmission of Crimean-Congo hemorrhagic fever virus by Hyalomma truncatum Koch. Am J Trop Med Hyg.

[B21] Sparagano OA, Allsopp MT, Mank RA, Rijpkema SG, Figueroa JV, Jongejan F (1999). Molecular detection of pathogen DNA in ticks (Acari: Ixodidae): a review. Exp Appl Acarol.

[B22] Zeller HG, Cornet JP, Camicas JL (1994). Experimental transmission of Crimean-Congo hemorrhagic fever virus by west African wild ground-feeding birds to Hyalomma marginatum rufipes ticks. Am J Trop Med Hyg.

[B23] Fagbami AH, Tomori O, Fabiyi A, Isoun TT (1975). Experimantal Congo virus (Ib -AN 7620) infection in primates. Virologie.

[B24] Swanepoel R, Leman PA, Burt FJ, Jardine J, Verwoerd DJ, Capua I, Bruckner GK, Burger WP (1998). Experimental infection of ostriches with Crimean-Congo haemorrhagic fever virus. Epidemiol Infect.

[B25] Fisher-Hoch SP, McCormick JB, Swanepoel R, Van Middlekoop A, Harvey S, Kustner HG (1992). Risk of human infections with Crimean-Congo hemorrhagic fever virus in a South African rural community. Am J Trop Med Hyg.

[B26] Mayers DL (1999). Exotic virus infections of military significance. Hemorrhagic fever viruses and pox virus infections. Dermatol Clin.

[B27] Vincent MJ, Sanchez AJ, Erickson BR, Basak A, Chretien M, Seidah NG, Nichol ST (2003). Crimean-Congo hemorrhagic fever virus glycoprotein proteolytic processing by subtilase SKI-1. J Virol.

[B28] Lenz O, ter Meulen J, Klenk HD, Seidah NG, Garten W (2001). The Lassa virus glycoprotein precursor GP-C is proteolytically processed by subtilase SKI-1/S1P. Proc Natl Acad Sci U S A.

[B29] Andersson AM, Melin L, Bean A, Pettersson RF (1997). A retention signal necessary and sufficient for Golgi localization maps to the cytoplasmic tail of a Bunyaviridae (Uukuniemi virus) membrane glycoprotein. J Virol.

[B30] Andersson AM, Pettersson RF (1998). Targeting of a short peptide derived from the cytoplasmic tail of the G1 membrane glycoprotein of Uukuniemi virus (Bunyaviridae) to the Golgi complex. J Virol.

[B31] Chen SY, Compans RW (1991). Oligomerization, transport, and Golgi retention of Punta Toro virus glycoproteins. J Virol.

[B32] Chen SY, Matsuoka Y, Compans RW (1991). Golgi complex localization of the Punta Toro virus G2 protein requires its association with the G1 protein. Virology.

[B33] Gerrard SR, Nichol ST (2002). Characterization of the Golgi retention motif of Rift Valley fever virus G(N) glycoprotein. J Virol.

[B34] Lappin DF, Nakitare GW, Palfreyman JW, Elliott RM (1994). Localization of Bunyamwera bunyavirus G1 glycoprotein to the Golgi requires association with G2 but not with NSm. J Gen Virol.

[B35] Matsuoka Y, Chen SY, Compans RW (1994). A signal for Golgi retention in the bunyavirus G1 glycoprotein. J Biol Chem.

[B36] Matsuoka Y, Chen SY, Holland CE, Compans RW (1996). Molecular determinants of Golgi retention in the Punta Toro virus G1 protein. Arch Biochem Biophys.

[B37] Pettersson RF, Andersson A, Melin L (1995). Mapping a retention signal for Golgi localization of a viral spike protein complex. Cold Spring Harb Symp Quant Biol.

[B38] Shi X, Lappin DF, Elliott RM (2004). Mapping the Golgi targeting and retention signal of Bunyamwera virus glycoproteins. J Virol.

[B39] Spiropoulou CF, Goldsmith CS, Shoemaker TR, Peters CJ, Compans RW (2003). Sin Nombre virus glycoprotein trafficking. Virology.

[B40] Flick R, Elgh F, Pettersson RF (2002). Mutational analysis of the Uukuniemi virus (Bunyaviridae family) promoter reveals two elements of functional importance. J Virol.

[B41] Flick R, Flick K, Feldmann H, Elgh F (2003). Reverse genetics for crimean-congo hemorrhagic fever virus. J Virol.

[B42] Mettenleiter TC (2002). Herpesvirus assembly and egress. J Virol.

[B43] Mackenzie JM, Jones MK, Westaway EG (1999). Markers for trans-Golgi membranes and the intermediate compartment localize to induced membranes with distinct replication functions in flavivirus-infected cells. J Virol.

[B44] Suzuki H (1996). A hypothesis about the mechanism of assembly of double-shelled rotavirus particles. Arch Virol Suppl.

[B45] Krijnse-Locker J, Ericsson M, Rottier PJ, Griffiths G (1994). Characterization of the budding compartment of mouse hepatitis virus: evidence that transport from the RER to the Golgi complex requires only one vesicular transport step. J Cell Biol.

[B46] Moss B, Knipe DMPMH (2001). Poxviridae: the viruses and their replication. Fields virology.

[B47] Hobman TC, Zhao B, Chan H, Farquhar MG (1998). Immunoisolation and characterization of a subdomain of the endoplasmic reticulum that concentrates proteins involved in COPII vesicle biogenesis. Mol Biol Cell.

[B48] Jackson MR, Nilsson T, Peterson PA (1990). Identification of a consensus motif for retention of transmembrane proteins in the endoplasmic reticulum. Embo J.

[B49] Hobman TC, Woodward L, Farquhar MG (1992). The rubella virus E1 glycoprotein is arrested in a novel post-ER, pre-Golgi compartment. J Cell Biol.

[B50] Hobman TC, Woodward L, Farquhar MG (1993). The rubella virus E2 and E1 spike glycoproteins are targeted to the Golgi complex. J Cell Biol.

[B51] Niwa H, Yamamura K, Miyazaki J (1991). Efficient selection for high-expression transfectants with a novel eukaryotic vector. Gene.

